# Infrared-sensing snakes select ambush orientation based on thermal backgrounds

**DOI:** 10.1038/s41598-019-40466-0

**Published:** 2019-03-08

**Authors:** Hannes A. Schraft, George S. Bakken, Rulon W. Clark

**Affiliations:** 1 Department of Biology, San Diego State University, San Diego, CA USA; 2 Graduate Group in Ecology, University of California, Davis, CA USA

## Abstract

Sensory information drives the ecology and behaviour of animals, and some animals are able to detect environmental cues unavailable to us. For example, rattlesnakes use infrared (IR) radiation to detect warm prey at night when visual cues are reduced. Until recently these sensory worlds have been inaccessible to human observers; now technology can allow us to “eavesdrop” on these species and understand how sensory perception drives ecology and behaviour. We used thermography and computer simulations to examine how prey-background temperature contrast and areas of temperature transitions influence the angular orientation of free-ranging rattlesnakes once they have selected an ambush site. We tracked free-ranging sidewinder rattlesnakes *Crotalus cerastes* to their selected ambush sites and recorded 360° near-ground thermographic panoramas from the centre of the ambush site. A computer simulation then moved a simulated prey item across the panorama and computed a contrast index for all directions. Rattlesnakes did not face ambush directions that offered stronger contrast than average, but they demonstrated a striking tendency to face directions with strong thermal transitions. Background transitions likely create a readily detected, rapidly changing stimulus when a prey animal passes. Quantifications of sensory environments like this one can boost our comprehension of how sensory function impacts the ecology, behaviour, and evolution of animals.

## Introduction

The sensory systems of some animals allow them to sense environmental stimuli that are not detectable by other organisms. These specialised sensory channels are often the foundation for ecological and behavioural specialisations and may be responsible for subsequent adaptive radiations. Conceptualizing these unfamiliar sensory worlds is difficult for human observers but is necessary for insight into fundamental questions in physiology, ecology, and behaviour. Advances in technology are now allowing human observes to access some of the sensory worlds of other species.

Rattlesnakes hunt by using highly developed chemosensory capabilities to locate areas of prey activity^1,2^. They then remain still and ambush animals that come within striking range. The predatory strike is guided by vision and another unique sense, the ability of pit vipers (Crotalinae, including rattlesnakes) to detect thermal infrared radiation (IR) invisible to humans and most other animals^3^. Here, we are interested in how pit vipers might use IR sensing to select a strike direction once a general area of prey activity has been located.

Pit vipers derive their common name from sensory facial pits located between the eyes and nostril. Each pit organ houses a suspended membrane covered with highly sensitive warm receptors (ca. 0.001 °C)^4^. Pit geometry creates a low resolution, pinhole-camera eye^3,5^ and IR information is integrated with visual information in the optic tectum^6,7^. IR detection is used for behavioural thermoregulation^8,9^ and to detect endothermic prey in the absence of light^10–12^. Experiments show that this enhanced sensory ability allows pit vipers to be more effective predators of nocturnal rodents compared to similar non-IR sensing vipers^13^.

Pit vipers primarily detect thermal contrast rather than absolute temperature^4,14,15^. The IR system essentially performs a ‘brightness constancy’ computation, analogous to how visual systems compare the luminance of a target to the background^15^. Optical and heat transfer analyses show that IR images on the pit membrane are likely quite blurry^16^, though some neural image sharpening and contrast enhancement may occur in the central nervous system^17–19^. The neuronal response of the infrared sensory system is phasic, i.e. it responds more to rate of change rather than the absolute level of a signal^4,20^.

The combination of thermal contrast detection, phasic neural response, and low-resolution imaging means that strong contrast and sudden changes in the thermal scene may be more easily detected than low contrast and slow changes. Thus, prey should be easier to locate and strike against thermal backgrounds that offer a strong, uniform contrast with prey animals^16,21^. In addition, prey animals might be more easily detected against thermal backgrounds with temperature transitions, i.e. areas where the contrast between moving prey and background changes rapidly, such that the prey animal will “pop” into view when it moves across the transition.

**Figure 1:**
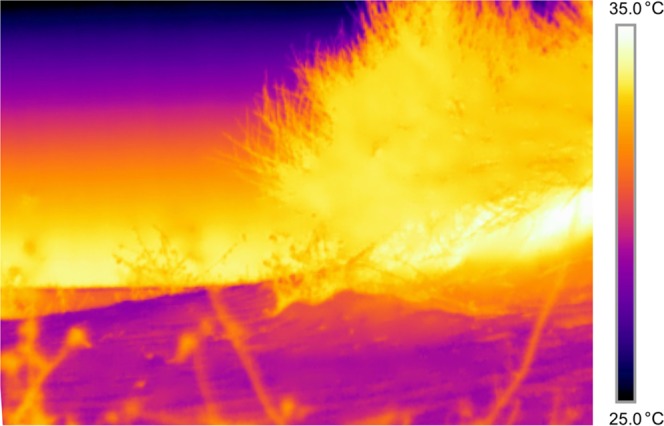
At night, bushes are typically warmer than the surrounding substrate, providing for varied thermal backgrounds. Image taken 2h50 after sunset with a FLIR T-420 thermal imaging camera (emissivity = 0.95, refl. temp. = 20 °C). Note that the images used in the analyses were of lower resolution (240 × 320 pixels).

Thermal backgrounds in the field are varied, offering options to hunting snakes. In night time desert habitat, shrubs and bushes are typically warmer than the surrounding open substrate due to micrometeorological patterns^22^, thus creating heterogenous thermal backgrounds (Fig. 1). After free-ranging nocturnal pit vipers use chemosensory cues to locate suitable hunting sites^1,2^, they may then orient towards backgrounds that optimise IR prey detection when settling into ambush.

However, to date we have almost no information on how IR detection is used in the natural environment. This shortcoming is critical because understanding the sensory worlds of other species can lead to unexpected insights into physics and physiology, findings which can sometimes be refined into unique technologies^23^. Advances in IR imaging have made it a useful tool in biological research^24^ and now provide the means to quantify many parameters relevant to IR sensing.

**Figure 2:**
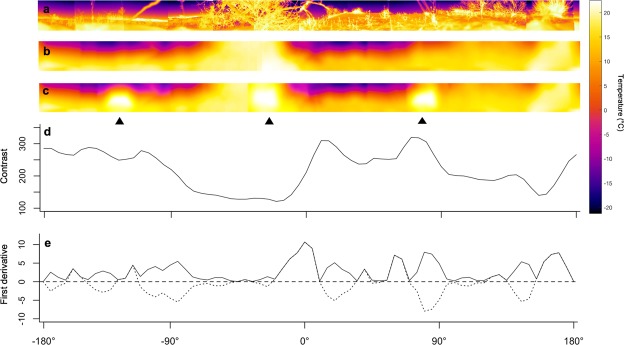
Overview of image processing and data extraction using a sample image. (**a**) 360° thermal panorama (240 × 4800 pixels) of a sample ambush site taken with a thermal imaging camera (original images 240 × 320 pixels each), (**b**) convoluted image as it might appear to a snake, (**c**) panorama with simulated kangaroo rat in three sample locations indicated by black triangles, (**d**) corresponding contrast for a simulated kangaroo rat moving across the panorama, and (**e**) rate of change (dotted line) and absolute value (solid line) of the rate of change of contrast (first derivative). Open areas have a higher contrast than areas with bushes; transitions between bushes and open areas have a high rate of change.

Here, we used radiometric thermography to record 360° IR panoramas surrounding ambush sites selected by sidewinder rattlesnakes, *Crotalus cerastes*. For each panorama, we then used a computer simulation to calculate a ‘contrast index’ of how detectable a rodent would be against 72 surrounding background points (Fig. 2). We then tested the hypothesis that snakes choose to face backgrounds that facilitate prey detection, i.e. areas of either strong contrast or thermal transitions.

## Results

We recorded 122 infrared panoramas from ambush positions selected by 67 individual snakes. The dataset includes a single panorama from 39 snakes, and two to eight panoramas from the remaining 28 snakes. Standard deviation of contrast values ranged from 4.1 to 99.3. Time since sunset was not a significant predictor of the variability (SD) in contrast values (r^2^ = −0.008, *p* = 0.947).

**Figure 3:**
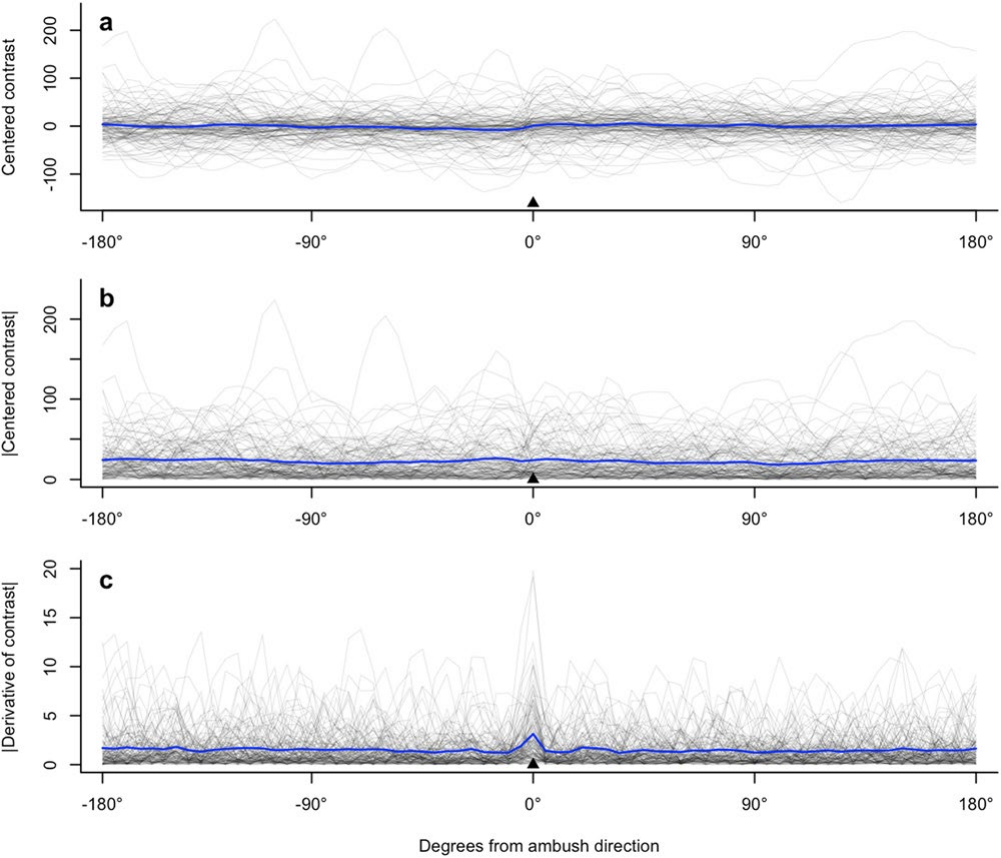
Contrast values and rate of change of contrast for all ambush sites, aligned using relative angles such that the snake is facing (ambush direction) 0° in all cases. Each line represents an ambush site; the blue line is the mean for all sites. (**a**) Raw contrast values for all ambush sites. Values are centred for plotting, so negative values here do not necessarily imply that the actual contrast was negative. (**b**) Absolute value of centred contrast values for all ambush sites. (**c**) Absolute value of rate of change of contrast values for all ambush sites.

Snakes did not face backgrounds that had higher contrast values than the average over the ambush site panorama (t = −0.84, *p* = 0.405; Table 1, Fig. 3b). However, snakes consistently faced backgrounds with a higher absolute rate of change of contrast values than the average (t = 5.28, *p* < 0.001; Table 1, Fig. 3c). The standard deviation of the random intercept for individual ID was low in both models (contrast model: 13.2; derivative of contrast model: 0.35), indicating that that there was low interindividual variability.

**Table 1:** Results of models comparing contrast or rate of change of contrast in ambush direction to mean panorama contrast or rate of change of contrast.

	Estimate	SE	df	t-value	*p*-value
***Contrast***					
Intercept	97.36	6.11	47.7	15.94	<0.001
Ambush direction contrast	−3.01	3.60	127.2	−0.84	0.405
***Rate of change of contrast***					
Intercept	1.66	0.19	86.3	8.85	<0.001
Ambush direction rate of change	1.04	0.20	160.1	5.28	<0.001

Both models include a random intercept for snake ID.

## Discussion

Prior studies suggest that foraging pit vipers should select cool, homogenous background temperatures. For example, a field study by Shine *et al*.^21^ found that arboreal ambush sites of Chinese pit vipers, *Gloydius shedaoensis* that prey on birds typically faced open sky, which presents low and relatively uniform background temperatures (Fig. 2)^25^. Also, laboratory studies have reported robust behavioural responses^14,15,26^ and higher strike success^15^ when thermal contrast between target and background was large and positive (target warmer than background). This preference for positive thermal contrast might be related to observations of antiphase tracking of moving pendulum targets with negative contrast^14,27^, which should be inimical to strike success.

Our results do not support the hypothesis that free-ranging sidewinder rattlesnakes orient to face backgrounds offering higher thermal contrast than the average at that ambush site. However, they did face directions that offered a higher rate of change in contrast than the average rate of change available at that site. This selectivity was highly significant and is readily apparent in Fig. 3c. This selection could be due to the phasic neural response of the pit organ^4,20^, which suggests that pit vipers may be particularly sensitive to the sudden change in signal when a potential prey animal crosses the boundary between areas with low contrast and high contrast, e.g. emerging into the open from under a bush. This response could be particularly important if the angular resolution of the pit organ is as low as theoretical analyses suggest^16^.

Because our results are based on observational data, it is possible that the apparent preference for thermal transitions, typically occurring at the edge of bushes, could be driven by other sensory cues. Bushes are often preferred rodent microhabitats as they provide cover (pers. obs.). Thus, ambush predators may face at or near bushes because other sensory cues (chemical, visual) are also used to locate sites frequented by prey. However, snakes in this study usually chose ambush sites in relatively open areas (approx. 1–5 m to nearest bush) clearly out of striking range of prey animals active in and near bushes, indicating that snakes did not simply seek out bushes.

Time constraints prevented us from testing whether snakes select ambush sites surrounded by more, or less, thermal variability than random sites. However, given the demonstrated importance of thermal backgrounds in prey capture^15^, we think it is likely that free-ranging pit vipers incorporate thermal characteristics of the environment into their selection of ambush sites, as well as using thermal characteristics to select strike directions within a site. Studies by Shine and colleagues^21,28^ indicate that this may be the case in Shedao pit vipers, *G*. *shedeaoensis*. Future studies should include field experiments using manipulated thermal backgrounds to examine the predation success of IR sensing and non-IR sensing snakes to definitively test these ideas.

The analysis developed for this study provides an example of how researchers can take advantage of emerging technologies and computational tools to quantify relevant parameters of an otherwise hidden sensory realm. We have provided one of the first quantitative and detailed analyses of the thermo-visual sensory world of free-ranging snakes, and we believe this approach will serve as an important foundation for further understanding of the unique sensory worlds inhabited by many animals.

## Methods

### Study site and animals

We recorded data at ambush sites selected by free-ranging sidewinder rattlesnakes, *Crotalus cerastes*, found on the Barry M. Goldwater range (managed by US Marine Corps) near Yuma, Arizona, USA. The most important mammalian prey of sidewinder rattlesnakes are kangaroo rats (*Dipodomys* spp.) followed by pocket mice (*Pergonathus* and *Chaetodipus* spp.)^29^. Nocturnal mammals are eaten by sidewinders ranging in size from ca. 25 to 60 cm snout-vent length (SVL)^29^. We collected data from snakes of all sizes, but our sample is skewed towards smaller snakes (SVL: 35.6 ± 5.6 cm, mean ± SD)^30^ because they were most abundant at our field site.

The study site consists of sand dunes interspersed with bushes^31^ (Fig. 1). Data collection took place between 19 May and 31 July 2017. During the summer months, sidewinders are primarily nocturnal^32^. Therefore, all data collection was performed in darkness between 20:00 h and 02:00 h, and in calm conditions to ensure consistent temperature gradients between sand and bushes. As thermal conditions and an individual’s direction of orientation may change over the night, we collected data (including thermal imaging) immediately after locating a snake in ambush posture to ensure ecological relevance. Although a time-lapse panorama of the ambush site and ambush direction would provide a more complete thermal picture, this was not logistically feasible.

### Field data collection

We located hunting snakes by following tracks left in the sand^33^. When rattlesnakes are ambush hunting, they locate a suitable site, stop, and assume a stereotypical coiled body position facing a particular direction^1,32^. We first recorded the compass bearing each snake was facing before temporarily collecting the snake. To record thermal panoramas, we centred an inverted tripod on the snake’s original head position and attached a FLIR T-420 thermal imaging camera (FLIR Systems, Wilsonville, OR, USA) to capture a series of near-ground thermal images later merged into a 360° thermal panorama (Fig. 2a; original image resolution 240 × 320 pixels). For all ambush sites, we positioned the camera to keep the horizon level and approximately in the middle of the image. Panoramas thus included, on average, 50% ground and 50% sky. In addition, we recorded air temperature (°C) and relative humidity (%) at each ambush site.

Snakes were measured, a microchip (8 mm PIT tags, Biomark, Boise, ID, USA) implanted for identification of recaptures, and released at the point of capture. We did occasionally recapture the same individuals later in the season, so we were able to collect data on more than one ambush site for some individuals. We recorded a total of 122 infrared panoramas from ambush sites selected by 67 individual snakes.

All methods were carried out in accordance with the relevant guidelines and regulations and approved by the San Diego State University Institutional Animal Care and Use Committee (APF 16-08-014 C).

### Data processing

We used FLIR Tools + v5.4.1 software (FLIR Systems, Wilsonville, OR, USA) to assemble and visually check the quality of all panoramas. Each panorama consisted of a 240 row x ca 4800 column matrix of temperatures (Fig. 2a). This was then imported into MATLAB R2017a (The MathWorks Inc., Natick, MA, USA).

The MATLAB script saved the panorama, and then generated a simulated rodent target roughly the size and shape of a Merriam’s kangaroo rat, *Dipodomys merriami*, viewed from 0.3 m. It consisted of two superimposed ellipses, one for the body and one for the head, with separate temperatures based on regression equations relating wild kangaroo rat surface temperatures to air temperature (data from^34^).

The script then placed target at one of 72 positions spaced at 5° intervals. The estimated appearance of the panorama to the snake was computed using the optical and heat transfer analysis procedure described in Bakken and Krochmal^16^ (Fig. 2b,c). We assumed an elliptical spread function (major diameter = 15°, minor diameter = 7.5°, air temperature as measured in field). This spread function is limited by the vertical angle of the thermal images. It is somewhat smaller and therefore would produce a slightly sharper image than the neurally sharpened forward direction spread function found by Stanford and Hartline^35^ for *C*. *oreganus*. Unfortunately, no data is available for the neurally sharpened spread function of sidewinders. The procedure was repeated for each placement of the target, creating 73 simulated panoramas for each ambush site, one without (Fig. 2b) and 72 with (Fig. 2c) the simulated rodent.

For each panorama, we then estimated how detectable the simulated rodent target might be against that background at each of the 72 evenly spaced compass directions as follows. Panoramas consisted of a matrix of pixels, each with an associated temperature value. To compute the contrast index of target detectability, we subtracted the image matrix without the target from the image matrix with the target. The result has zeros for all pixels except those where the target is located. There, the value is the difference between background and target temperature. We then summed over rows and columns to compute a contrast index equal to the area x temperature difference for each rodent position around the panorama (Fig. 2d; Supplementary Video; MATLAB script available on request). We refer to this measure as ‘contrast’, where higher values indicate a greater total temperature change caused by the presence of the rodent. In addition, we calculated the rate of change (first derivative) of contrast values across a panorama to identify points where this contrast index changed rapidly (Fig. 2e). The sign of the first derivative of contrast only indicates the direction of simulated motion, so absolute values were used for analysis.

### Statistical analysis

We used linear mixed models to determine whether contrast or rate of change of contrast in ambush direction were different from the contrast or rate of change of contrast averaged over the entire panorama. This allowed us to determine if snakes chose to face ambush directions with higher thermal contrast or higher rate of contrast change than available on average. The models included a random intercept for snake ID to statistically account for the non-independence of repeated measurements from the same snake. Snakes respond to positive and negative thermal contrast, though response to negative contrast is typically weaker^14,15,26^. We therefore analysed absolute contrast values. There was considerable variability among panoramas in the range of contrast values. This could be problematic as the direction faced is probably irrelevant to snakes when contrast is nearly uniform. We therefore weighted panoramas according to within-panorama standard deviation of raw contrast values.

The qualitative properties of the thermal environment may change over the course of the night as bushes, air, and substrate temperatures cool at different rates. Therefore, in the absence of time lapse data, we ran a linear regression to test whether the variability in contrast values was a function of time since sunset. All statistical analyses were done in *R* v3.2.3^36^ using the package *lme4*^37^.

## Supplementary information

Supplementary video

Analysis

Dataset 1

Dataset 2

## Data Availability

Original data and *R* script for analysis and figures are attached as Supplementary Information.
