# Supplementary material for: Infrared-sensing snakes select ambush orientation based on thermal backgrounds
Source: Sci Rep. 2019 Mar 8;9:3950. doi: 10.1038/s41598-019-40466-0 (PMC6408448; doi:10.1038/s41598-019-40466-0)
Supplement: Supplementary file 2 — Analysis [file 41598_2019_40466_MOESM2_ESM.pdf]

Infrared-sensing snakes select ambush orientation based on thermal backgrounds

Hannes Schraft, George Bakken, Rulon Clark

Contents

1 Data wrangling **1**

2 Data overview **2**
2.1 Sample size 2
2.2 Number of times that each snake was tested 2
2.3 Range of standard deviation of contrast values 4

3 Figures **5**

4 Models **5**
4.1 Contrast model 7
4.2 Derivatives model 8
4.3 Does time since sunset affect the thermal heterogeneity of panoramas? 8

1 Data wrangling

```
# read in data, retaining row names
contrast.raw <- read.csv("contrasts.csv", row.names = 1)

# remove column names
colnames(contrast.raw) <- NULL

# display first 6 rows and first 6 columns of raw data
contrast.raw[1:6, 1:6]
```

```
##
## trial07  239.470 232.500 227.490 227.820 233.400 230.840
## trial08  299.110 265.250 248.670 245.810 245.410 242.630
## trial09  378.990 387.690 343.670 299.400 248.920 264.080
## trial100  33.584  30.296  41.982  57.137  69.820  62.714
## trial101  87.825  84.586  77.355  66.369  60.813  66.352
## trial102  80.459  74.841  74.720  72.643  51.949  41.282
```

These are the raw contrast values produced by the MATLAB routine. Each row is a different panorama; columns contain the contrast values in different directions. Ambush direction is in between columns 36 and 37 in each panorama.

There is hidden code to…

- Calculate the within-panorama standard deviation of contrast values, for use as the 'weights' argument in the models
- Take the absolute value of all contrast values, because both large positive and large negative contrasts might be of interest to snakes
- For each panorama, calculate the mean contrast and the contrast in ambush direction

- Calculate the first derivative across each panorama, followed by the mean derivative of the entire panorama and the derivative in ambush direction
- Import and attach trial metadata

To view it, please open the .rmd file in R or R Studio.

2 Data overview

This is the analysis dataset:

```
# display first six rows of 'analysisdata'
head(analysisdata)
```

```
##          trialID snakeID  SVL time.since.sunset       SD mean.contrast
## trial07        7  snake7 45.9               287 63.03541     157.13012
## trial08        8  snake8 25.1                75 68.34004     188.11596
## trial09        9  snake9 37.7               118 61.06035     258.27347
## trial100     100 snake33 39.8               105 19.69473      60.78940
## trial101     101 snake62 38.0               123 74.34459     109.53045
## trial102     102 snake62 38.0               145 32.74705      89.04707
##          ambush.contrast mean.derivative ambush.derivative
## trial07          79.0000        1.478617            0.6034
## trial08         167.3600        3.697122            4.0610
## trial09         210.9350        3.470833            6.8920
## trial100         23.0105        1.227589            0.4813
## trial101         72.6110        3.956954           13.3172
## trial102         88.2650        2.261444            0.2440
```

2.1 Sample size

The sample size is. . .

```
# display number of trials
length(unique(analysisdata$trialID))
```

```
## [1] 122
```

. . . trials, on. . .

```
# display number of snakes
length(unique(analysisdata$snakeID))
```

```
## [1] 67
```

. . . individual snakes.

2.2 Number of times that each snake was tested

```
# count number times that each snake was tested
print(count(analysisdata, snakeID), n = Inf)
```

```
## # A tibble: 67 x 2
##    snakeID     n
```

```
##      <fctr> <int>
##   1  snake1     1
##   2 snake10     4
##   3 snake11     3
##   4 snake12     1
##   5 snake13     1
##   6 snake14     1
##   7 snake15     5
##   8 snake16     3
##   9 snake17     1
##  10 snake18     2
##  11 snake19     1
##  12  snake2     3
##  13 snake20     4
##  14 snake21     2
##  15 snake23     2
##  16 snake24     1
##  17 snake25     1
##  18 snake26     1
##  19 snake27     3
##  20 snake28     1
##  21 snake29     1
##  22 snake30     1
##  23 snake31     1
##  24 snake32     4
##  25 snake33     3
##  26 snake34     1
##  27 snake35     1
##  28 snake36     1
##  29 snake37     1
##  30 snake38     1
##  31 snake39     3
##  32  snake4     3
##  33 snake40     2
##  34 snake41     2
##  35 snake42     3
##  36 snake43     1
##  37 snake44     1
##  38 snake45     1
##  39 snake46     2
##  40 snake47     1
##  41 snake49     1
##  42  snake5     1
##  43 snake50     3
##  44 snake51     2
##  45 snake52     3
##  46 snake53     1
##  47 snake54     2
##  48 snake55     1
##  49 snake56     1
##  50 snake57     2
##  51 snake58     3
##  52 snake59     1
##  53 snake62     3
```

```
## 54 snake63     1
## 55 snake64     1
## 56 snake65     1
## 57  snake7     1
## 58 snake70     1
## 59 snake71     1
## 60 snake72     1
## 61 snake73     1
## 62 snake79     2
## 63  snake8     1
## 64 snake80     1
## 65 snake81     2
## 66 snake84     1
## 67  snake9     8
```

```
counts <- count(analysisdata, snakeID)

# display how many snakes were tested N number of times
sum(counts$n == '1')
```

```
## [1] 39
```

```
sum(counts$n == '2')
```

```
## [1] 11
```

```
sum(counts$n == '3')
```

```
## [1] 12
```

```
sum(counts$n == '4')
```

```
## [1] 3
```

```
sum(counts$n == '5')
```

```
## [1] 1
```

```
sum(counts$n == '6')
```

```
## [1] 0
```

```
sum(counts$n == '7')
```

```
## [1] 0
```

```
sum(counts$n == '8')
```

```
## [1] 1
```

2.3 Range of standard deviation of contrast values

```
range(analysisdata$SD)
```

```
## [1]  4.059362 99.279400
```

3 Figures

Open the .rmd file to view the code to produce the figures.

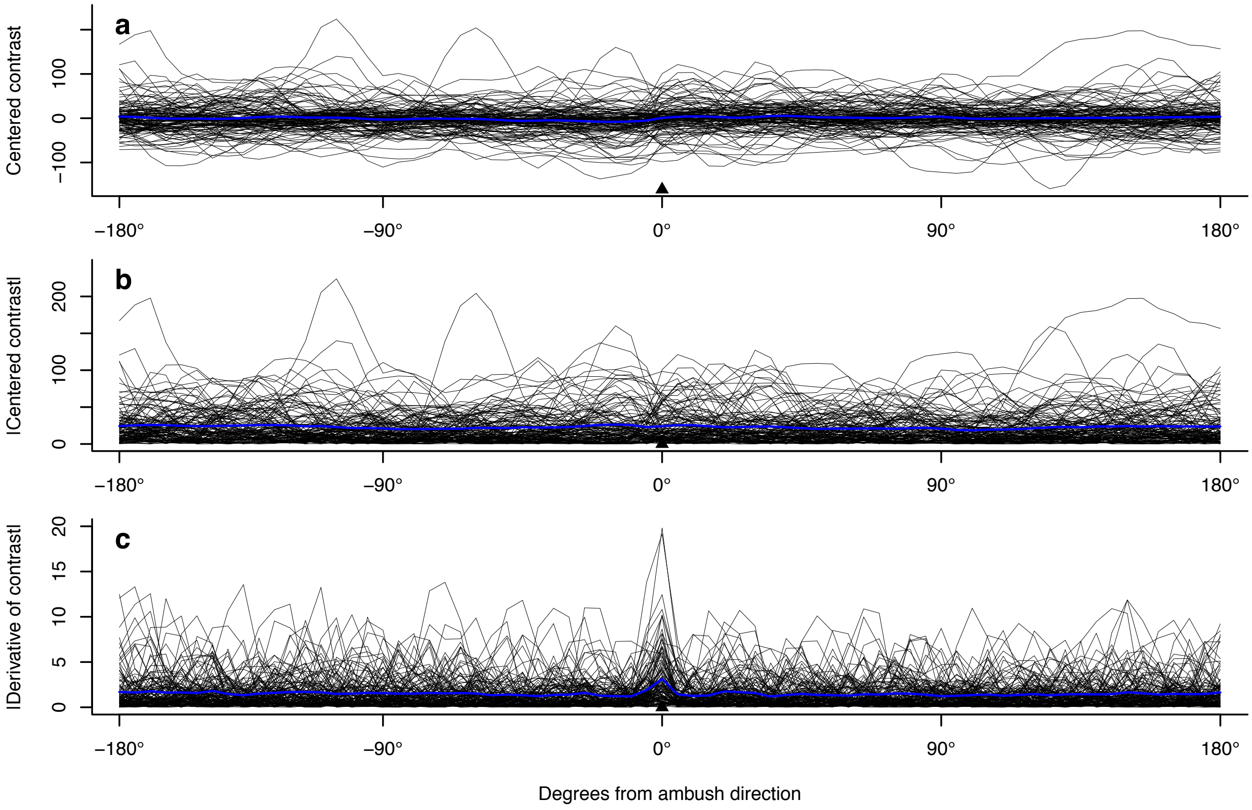

4 Models

This is the basic model structure:

```
contrast ~ contrast.type + (1|trialID) + (1|snakeID)
```

Where *contrast* is the values calculated by the MATLAB routine. *contrast.type* indicates whether *contrast* refers to the contrast in the ambush direction or contrast averaged over the entire panorama. The random effect for *trialID* ensures that the model compares ambush contrasts and mean contrasts within a given panorama. The random effect for *snakeID* accounts for the fact that there are multiple panoramas from some snakes. *trialID* is automatically nested within *snakeID*.

Right now the data are in wide format:

```
# display first five rows of 'analysisdata'
analysisdata[1:5, ]
```

```
##          trialID snakeID  SVL time.since.sunset       SD mean.contrast
## trial07        7  snake7 45.9               287 63.03541      157.1301
## trial08        8  snake8 25.1                75 68.34004      188.1160
## trial09        9  snake9 37.7               118 61.06035      258.2735
## trial100     100 snake33 39.8               105 19.69473       60.7894
## trial101     101 snake62 38.0               123 74.34459      109.5304
##          ambush.contrast mean.derivative ambush.derivative
```

```
## trial07          79.0000        1.478617            0.6034
## trial08         167.3600        3.697122            4.0610
## trial09         210.9350        3.470833            6.8920
## trial100         23.0105        1.227589            0.4813
## trial101         72.6110        3.956954           13.3172
```

To use the above model structure they need to be in long format.

```
# make long dataset for contrast model
contrasts.long <- gather(analysisdata,
                         contrast.type,
                         contrast,
                         ambush.contrast,
                         mean.contrast,
                         factor_key = TRUE)

# sort by trialID and snakeID
contrasts.long <- contrasts.long[order(contrasts.long$trialID,
                                       contrasts.long$snakeID), ]

# display first five rows of 'contrasts.long'
contrasts.long[1:5, ]
```

```
##     trialID snakeID  SVL time.since.sunset       SD mean.derivative
## 1         7  snake7 45.9               287 63.03541        1.478617
## 123       7  snake7 45.9               287 63.03541        1.478617
## 2         8  snake8 25.1                75 68.34004        3.697122
## 124       8  snake8 25.1                75 68.34004        3.697122
## 3         9  snake9 37.7               118 61.06035        3.470833
##     ambush.derivative   contrast.type contrast
## 1              0.6034 ambush.contrast  79.0000
## 123            0.6034   mean.contrast 157.1301
## 2              4.0610 ambush.contrast 167.3600
## 124            4.0610   mean.contrast 188.1160
## 3              6.8920 ambush.contrast 210.9350
```

```
# make long dataset for derivatives model
derivatives.long <- gather(analysisdata,
                           derivative.type,
                           derivative,
                           ambush.derivative,
                           mean.derivative,
                           factor_key = TRUE)

# sort by trialID and snakeID
derivatives.long <- derivatives.long[order(derivatives.long$trialID,
                                           derivatives.long$snakeID), ]

# display first five rows of 'derivatives.long'
derivatives.long[1:5, ]
```

```
##     trialID snakeID  SVL time.since.sunset       SD mean.contrast
## 1         7  snake7 45.9               287 63.03541      157.1301
## 123       7  snake7 45.9               287 63.03541      157.1301
## 2         8  snake8 25.1                75 68.34004      188.1160
## 124       8  snake8 25.1                75 68.34004      188.1160
```

```
## 3         9  snake9 37.7               118 61.06035      258.2735
##     ambush.contrast   derivative.type derivative
## 1            79.000 ambush.derivative   0.603400
## 123          79.000   mean.derivative   1.478617
## 2           167.360 ambush.derivative   4.061000
## 124         167.360   mean.derivative   3.697122
## 3           210.935 ambush.derivative   6.892000
```

4.1 Contrast model

```r
# relevel contrast.type factor so that mean.contrast is the reference level
contrasts.long <- within(contrasts.long,
                         contrast.type <- relevel(contrast.type,
                                                  ref = "mean.contrast"))
```

```r
m.contrast <- lmer(contrast ~ contrast.type + (1|trialID) + (1|snakeID),
                   weights = SD,
                   data = contrasts.long)

summary(m.contrast)
```

```
## Linear mixed model fit by REML t-tests use Satterthwaite approximations
##   to degrees of freedom [lmerMod]
## Formula: contrast ~ contrast.type + (1 | trialID) + (1 | snakeID)
##    Data: contrasts.long
## Weights: SD
##
## REML criterion at convergence: 2614
##
## Scaled residuals:
##     Min      1Q  Median      3Q     Max
## -2.2253 -0.4416 -0.1851  0.2493  2.6044
##
## Random effects:
##  Groups   Name        Variance Std.Dev.
##  trialID  (Intercept)  3117.9   55.84
##  snakeID  (Intercept)   174.3   13.20
##  Residual             22208.8  149.03
## Number of obs: 244, groups:  trialID, 122; snakeID, 67
##
## Fixed effects:
##                              Estimate Std. Error      df t value Pr(>|t|)
## (Intercept)                    97.358      6.108  47.740  15.939   <2e-16
## contrast.typeambush.contrast   -3.006      3.600 127.220  -0.835    0.405
##
## (Intercept)                  ***
## contrast.typeambush.contrast
## ---
## Signif. codes:  0 '***' 0.001 '**' 0.01 '*' 0.05 '.' 0.1 ' ' 1
##
## Correlation of Fixed Effects:
##             (Intr)
## cntrst.typ. -0.295
```

4.2 Derivatives model

```r
# relevel derivative.type factor so that mean.derivative is reference level
derivatives.long <- within(derivatives.long,
                           derivative.type <- relevel(derivative.type,
                                                      ref = "mean.derivative"))
```

```r
m.derivative <- lmer(derivative ~ derivative.type + (1|trialID) + (1|snakeID),
                     weights = SD,
                     data = derivatives.long)

summary(m.derivative)
```

```
## Linear mixed model fit by REML t-tests use Satterthwaite approximations
##   to degrees of freedom [lmerMod]
## Formula: derivative ~ derivative.type + (1 | trialID) + (1 | snakeID)
##    Data: derivatives.long
## Weights: SD
##
## REML criterion at convergence: 1047
##
## Scaled residuals:
##     Min      1Q  Median      3Q     Max
## -2.7804 -0.4978 -0.2059  0.1775  6.0303
##
## Random effects:
##  Groups   Name        Variance Std.Dev.
##  trialID  (Intercept)  1.3417  1.1583
##  snakeID  (Intercept)  0.1201  0.3466
##  Residual             66.3316  8.1444
## Number of obs: 244, groups:  trialID, 122; snakeID, 67
##
## Fixed effects:
##                                 Estimate Std. Error       df t value
## (Intercept)                       1.6624     0.1879  86.2900   8.848
## derivative.typeambush.derivative   1.0379     0.1967 160.0700   5.275
##                                 Pr(>|t|)
## (Intercept)                     9.77e-14 ***
## derivative.typeambush.derivative 4.24e-07 ***
## ---
## Signif. codes:  0 '***' 0.001 '**' 0.01 '*' 0.05 '.' 0.1 ' ' 1
##
## Correlation of Fixed Effects:
##             (Intr)
## drvtv.typm. -0.524
```

4.3 Does time since sunset affect the thermal heterogeneity of panoramas?

```r
m.sunset <- lm(SD ~ time.since.sunset, data = analysisdata)

summary(m.sunset)
```

```
##
```

```
## Call:
## lm(formula = SD ~ time.since.sunset, data = analysisdata)
##
## Residuals:
##     Min      1Q  Median      3Q     Max
## -24.153 -15.235  -1.872   9.270  71.080
##
## Coefficients:
##                    Estimate Std. Error t value Pr(>|t|)
## (Intercept)       28.293288   3.377073   8.378 1.18e-13 ***
## time.since.sunset -0.001178   0.017741  -0.066    0.947
## ---
## Signif. codes:  0 '***' 0.001 '**' 0.01 '*' 0.05 '.' 0.1 ' ' 1
##
## Residual standard error: 17.68 on 120 degrees of freedom
## Multiple R-squared:  3.675e-05,  Adjusted R-squared:  -0.008296
## F-statistic: 0.004411 on 1 and 120 DF,  p-value: 0.9472
```

COPY-PASTE INTO R-STUDIO:

````
---
title: "Infrared-sensing snakes select ambush orientation based
on thermal backgrounds"
author: "Hannes Schraft, George Bakken, Rulon Clark"
date: ""
output:
  pdf_document:
    toc: true
    number_sections: true
editor_options:
  chunk_output_type: console
---

\
\
```{r setup, include = FALSE}

knitr::opts_chunk$set(echo = TRUE)

library(tidyr) # for data wrangling
library(dplyr) # for data wrangling
library(lme4) # for running LMMs
library(lmerTest) # for summarizing LMMs

```

# Data wrangling

```{r import data}

# read in data, retaining row names
contrast.raw <- read.csv("contrasts.csv", row.names = 1)

# remove column names
colnames(contrast.raw) <- NULL

# display first 6 rows and first 6 columns of raw data
contrast.raw[1:6, 1:6]

```

These are the raw contrast values produced by the MATLAB
routine. Each row is a different panorama; columns contain the
contrast values in different directions. Ambush direction is in
between columns 36 and 37 in each panorama.

There is hidden code to...

* Calculate the within-panorama standard deviation of contrast
values, for use as the 'weights' argument in the models
````

* Take the absolute value of all contrast values, because both large positive and large negative contrasts might be of interest to snakes
* For each panorama, calculate the mean contrast and the contrast in ambush direction
* Calculate the first derivative across each panorama, followed by the mean derivative of the entire panorama and the derivative in ambush direction
* Import and attach trial metadata

To view it, please open the .rmd file in R or R Studio.

```{r calculate panorama SD, echo = FALSE}

# calculate within-panorama standard deviation. This will be used in "weights" argument in the models later
SD <- apply(contrast.raw, 1, sd)

```

```{r take absolute value, echo = FALSE}

# take absolute value of all contrast values
contrast.abs <- abs(contrast.raw)

```

```{r calculate overall and ambush mean, echo = FALSE}

# calculate mean contrast of entire panorama
mean.contrast <- apply(contrast.abs, 1, mean)

# take the mean of columns 36 and 37 (ambush direction is at index = 36.5 right now)
ambush.contrast <- apply(contrast.abs[, 36:37], 1, mean)

```

```{r calculate derivatives, echo = FALSE}

# convert to matrix. Otherwise for-loop struggles
contrast.raw.m <- as.matrix(contrast.raw)

# paste last column to beginning of dataframe to end up with 72 difference values (instead of 71)
contrast.diff <- cbind(contrast.raw.m[, 72], contrast.raw.m)

# make sequence for x values (contrast values are the y values)
diff.seq <- seq(from = 0, to = 360, by = 5)

# create empty list to store results of for-loop in
diff.list <- list()

for (i in 1:nrow(contrast.diff)) {
```

````
  derivatives <- diff(contrast.diff[i, ])/diff(diff.seq)
  diff.list[[i]] <- abs(derivatives)
}

# convert list to dataframe
contrast.derivatives <- do.call("rbind", diff.list)

# attach row names
rownames(contrast.derivatives) <- rownames(contrast.diff)

# remove column names
colnames(contrast.derivatives) <- NULL

```

```{r calculate overall and ambush derivatives, echo = FALSE}

# calculate mean derivative of entire panorama
mean.derivative <- apply(contrast.derivatives, 1, mean)

# take the mean of columns 36 and 37 (ambush direction is at
index = 36.5 right now)
ambush.derivative <- apply(contrast.derivatives[, 36:37], 1,
mean)

```

```{r import metadata, echo = FALSE}

# load separate dataframe with trial meta data
metadata <- read.csv("metadata.csv")

# make analysis dataset
analysisdata <- cbind(metadata, SD, mean.contrast,
ambush.contrast, mean.derivative, ambush.derivative)

```
\


# Data overview


This is the analysis dataset:

```{r head of analysis dataset}

# display first six rows of 'analysisdata'
head(analysisdata)

```


## Sample size
````
````

The sample size is...

```{r display sample size - number of trials}

# display number of trials
length(unique(analysisdata$trialID))

```
\

...trials, on...

```{r display sample size - number of snakes}

# display number of snakes
length(unique(analysisdata$snakeID))

```

...individual snakes.
\

Number of times that each snake was tested

```{r count number times that each snake was tested}

# count number times that each snake was tested
print(count(analysisdata, snakeID), n = Inf)

counts <- count(analysisdata, snakeID)

# display how many snakes were tested N number of times
sum(counts$n == '1')
sum(counts$n == '2')
sum(counts$n == '3')
sum(counts$n == '4')
sum(counts$n == '5')
sum(counts$n == '6')
sum(counts$n == '7')
sum(counts$n == '8')

```

Range of standard deviation of contrast values

```{r range of SD}

range(analysisdata$SD)

```

Figures

Open the .rmd file to view the code to produce the figures.

````
```{r centering contrast values for making figures, echo =
FALSE}

# center raw contrast values
contrast.c <- t(apply(contrast.raw, 1, function(x) scale(x,
center = TRUE, scale = FALSE)))

# take absolute value for Fig 3B
abs.contrast.c <- abs(contrast.c)

```
````

\

````
```{r fig 3 for MS, echo = FALSE, eval = TRUE, warning = FALSE,
fig.cap = "**Manuscript Figure 3**", fig.height = 5, fig.width =
8}

par(mfrow = c(3, 1))

par(mar = c(1, 5, 1, 2) + 0.1)
par(fig = c(0, 1, 0.7, 1), new = TRUE)
par(plt = c(0.1, 0.95, 0.2, 0.95))
par(bty = "l")
plot(NULL,
     xlim = c(2, 71),
     ylim = c(-160, 240),
     ylab = "Centered contrast",
     xaxt = "n",
     xlab = ""
)

# plot the centered contrast values for each panorama
for(i in 1:nrow(contrast.c)) {
  line <- contrast.c[i,]
  lines(line, lwd = 0.07)
}
points(36, -161, pch = 17, cex = 1.2 )
axis(side = 1,
     at = c(((1/72)*72),
            (0.25*72),
            (0.5*72),
            (0.75*72),
            (1*72)),
     labels = c("-180°","-90°","0°","90°","180°"))
means <- colMeans(contrast.c)
lines(means, lwd = 1.3, col = "blue")
mtext(paste0("a"), side = 3, adj = 0.02, line = -1.5, font = 2)
````
````

```
# plot the absolute values of centered contrast values
par(mar = c(1, 5, 0.5, 2) + 0.1)
par(fig = c(0, 1, 0.4, 0.7), new = TRUE)
par(plt = c(0.1, 0.95, 0.2, 0.95))
plot(NULL,
     xlim = c(2, 71),
     ylim = c(0, 240),
     ylab = "|Centered contrast|",
     xaxt = "n",
     yaxt = "n",
     xlab = ""
)
for(i in 1:nrow(abs.contrast.c)) {
  line <- abs.contrast.c[i,]
  lines(line, lwd = 0.07)
}
points(36, 0, pch = 17, cex = 1.2 )
axis(side = 1,
     at = c(((1/72)*72),
            (0.25*72),
            (0.5*72),
            (0.75*72),
            (1*72)),
     labels = c("-180°","-90°","0°","90°","180°"))
axis(side = 2,
     at = c(0, 50, 100, 150, 200),
     labels = c("0", "", "100", "", "200"))
means <- colMeans(abs.contrast.c)
lines(means, lwd = 1.3, col = "blue")
mtext(paste0("b"), side = 3, adj = 0.02, line = -1.5, font = 2)

# plot absolute value of first derivative
par(mar = c(5, 5, 0.5, 2) + 0.1)
par(fig = c(0, 1, 0, 0.4), new = TRUE)
par(plt = c(0.1, 0.95, 0.375, 0.95))
plot(NULL,
     xlim = c(2, 71),
     ylim = c(0, 20),
     ylab = "|Derivative of contrast|",
     xaxt = "n",
     xlab = "Degrees from ambush direction"
)
for(i in 1:nrow(contrast.derivatives)) {
  line <- contrast.derivatives[i,]
  lines(line, lwd = 0.07)
}
points(36, 0, pch = 17, cex = 1.2 )
axis(side = 1,
     at = c(((1/72)*72),
            (0.25*72),
            (0.5*72),
            (0.75*72),
            (1*72)),
     labels = c("-180°","-90°","0°","90°","180°"))
```

```
means <- colMeans(contrast.derivatives)
lines(means, lwd = 1.3, col = "blue")
mtext(paste0("c"), side = 3, adj = 0.02, line = -1.5, font = 2)
```

\

Models

This is the basic model structure:

```{r, eval = FALSE}

contrast ~ contrast.type + (1|trialID) + (1|snakeID)

```

Where *contrast* is the values calculated by the MATLAB routine. *contrast.type* indicates whether *contrast* refers to the contrast in the ambush direction or contrast averaged over the entire panorama. The random effect for *trialID* ensures that the model compares ambush contrasts and mean contrasts within a given panorama. The random effect for *snakeID* accounts for the fact that there are multiple panoramas from some snakes. *trialID* is automatically nested within *snakeID*.

Right now the data are in wide format:

```{r display head of analysisdata, echo = TRUE}

# display first five rows of 'analysisdata'
analysisdata[1:5, ]

```

\
\

To use the above model structure they need to be in long format.

```{r convert contrasts to long format}

# make long dataset for contrast model
contrasts.long <- gather(analysisdata,
                         contrast.type,
                         contrast,
                         ambush.contrast,
                         mean.contrast,
                         factor_key = TRUE)

# sort by trialID and snakeID
contrasts.long <- contrasts.long[order(contrasts.long$trialID,
```

````
                                       contrasts.long$snakeID),
]

# display first five rows of 'contrasts.long'
contrasts.long[1:5, ]

```
\

```{r convert derivatives to long format, echo = TRUE}

# make long dataset for derivatives model
derivatives.long <- gather(analysisdata,
                           derivative.type,
                           derivative,
                           ambush.derivative,
                           mean.derivative,
                           factor_key = TRUE)

# sort by trialID and snakeID
derivatives.long <-
derivatives.long[order(derivatives.long$trialID,

derivatives.long$snakeID), ]

# display first five rows of 'derivatives.long'
derivatives.long[1:5, ]

```



## Contrast model

```{r relevel contrast factor, echo = TRUE}

# relevel contrast.type factor so that mean.contrast is the
reference level
contrasts.long <- within(contrasts.long,
                         contrast.type <- relevel(contrast.type,
                                                  ref =
"mean.contrast"))

```


```{r contrast model}

m.contrast <- lmer(contrast ~ contrast.type + (1|trialID) +
(1|snakeID),
                   weights = SD,
                   data = contrasts.long)

summary(m.contrast)

```
````
````

Derivatives model

```{r relevel derivative factor}

# relevel derivative.type factor so that mean.derivative is
reference level
derivatives.long <- within(derivatives.long,
                           derivative.type <-
relevel(derivative.type,
                                                    ref =
"mean.derivative"))

```

```{r derivative model}

m.derivative <- lmer(derivative ~ derivative.type + (1|trialID)
+ (1|snakeID),
                     weights = SD,
                     data = derivatives.long)

summary(m.derivative)

```


Does time since sunset affect the thermal heterogeneity of
panoramas?

```{r sunset model}

m.sunset <- lm(SD ~ time.since.sunset, data = analysisdata)

summary(m.sunset)

```
